# Epidural Hematoma related to lower limb pain and massive liver bleeding in Gorham-Stout disease: A case report

**DOI:** 10.1097/MD.0000000000033950

**Published:** 2023-06-02

**Authors:** Yasutomo Kumakura, Norio Hasuda, Kazuki Akita, Tetsuya Iijima, Takashi Matsukawa

**Affiliations:** a Department of Anesthesiology, Faculty of Medicine, University of Yamanashi, Chuo, Japan; b Department of Surgery 2, Faculty of Medicine, University of Yamanashi, Chuo, Japan.

**Keywords:** bleeding, cerebrospinal fluid leak, coagulation disorders, epidural hematoma, Gorham-Stout disease

## Abstract

Gorham-Stout disease (GSD) is a rare disease that causes massive osteolysis and proliferation of abnormal lymphangiomatous tissues. Patients with GSD often experience pain associated with bone fractures and chylothorax. However, bleeding caused by abnormal lymphangiomatous tissue or hematological dysfunction rarely occurs.

**Patient concerns:**

A 22-year-old female patient with GSD presented with severe left hip and lower limb pain. The GSD had disappeared her right pelvic bone and femur, but no abnormalities were found in the bones at the site of the pain.

**Diagnoses:**

The patient presented with a chylothorax and cerebrospinal fluid leakage. She was treated with sirolimus and an epidural blood patch, and her symptoms resolved. Computed tomography and magnetic resonance imaging revealed an epidural hematoma extending from L3 to the caudal region, and blood results revealed a consumption coagulopathy.

**Interventions:**

We presumed that the hematoma caused pain and prescribed pregabalin and morphine. The pain gradually subsided.

**Outcomes:**

An unexpected liver subcapsular hemorrhage occurred 4 months later, and the patient went into hemorrhagic shock. Transcatheter arterial embolization was promptly performed, and the patient recovered.

**Lessons:**

GSD infrequently causes bleeding related to abnormal lymphangiomatous tissues and coagulopathy, yet it can lead to serious events if it occurs.

## 1. Introduction

Gorham-Stout disease (GSD), often called “Vanishing bone disease,” is a rare disease characterized by the progression of massive osteolysis and the proliferation of lymphangiomatous tissues. The condition of massive idiopathic osteolysis was first described by Jackson in 1838,^[1]^ and LW Gorham and AP Stout reported that progressive osteolysis was associated with angiomatoses of the blood and lymphatic vessels.^[2]^

It has been reported that GSD is not associated with race or sex.^[3]^ GSD most commonly affects children and young adults, with an average age at diagnosis of 25 years.^[4,5]^ The etiology of GSD is unclear. Some studies have suggested that interleukin-6, macrophage colony-stimulating factor, vascular endothelial growth factor (VEGF), and receptor activator of NF-kappaB ligand play a crucial role in osteoclast function and promoting lymphangiogenesis.^[6,7]^ GSD may involve any bone of the whole body, but it is commonly found in the upper part of the body, vertebrae, ribs, and pelvic bones.

The most common symptom of GSD is localized pain in osteolytic lesions.^[8,9]^ Patients with GSD commonly experience osteolysis and a decline in activities of daily living, which leads to pathological fractures. However, the proliferation of lymphangiomatous tissue around the affected lesion can cause various complications, including cerebrospinal fluid (CSF) leakage and chylothorax, leading to symptoms such as headache and dyspnea.^[10]^

In this report, we describe a case in which proliferating lymphangiomatous tissue in the spinal canal caused severe headache and lower limb pain in a patient with GSD. Consumption coagulopathy may lead to bleeding.

## 2. Case presentation

A 22-year-old female patient with GSD presented with severe pain in the left hip, lower limb, and head. She was diagnosed with GSD based on an osteolytic lesion biopsied at 12 years of age. She experienced right lower limb osteolysis at 2 years of age, which gradually progressed to the right femur, pelvic bone, and lumbar vertebrae. Despite treatment with interferon-alpha, bisphosphonate, and vitamin D, the disease progressed, and the patient’s condition worsened. At 14 years of age, the patient presented with a pleural effusion and chylothorax. Pleurodesis was performed, and sirolimus was administered, following which fluid accumulation in the pleura decreased. However, the osteolytic lesions gradually extended into the sacrum. At 18 years of age, the patient complained of severe orthostatic headaches. Examination revealed a recurrent bilateral acute subdural hematoma. Radioisotope inspection showed CSF leakage and accumulation in the lymphangiomatous tissue at the dura mater of L3; thus, she was diagnosed with cerebrospinal fluid hypovolemia due to a CSF-lymph fistula. After multiple burr hole irrigation and drainage, an epidural blood patch was performed. The dyspnea and headache gradually disappeared.

**Figure 1.**
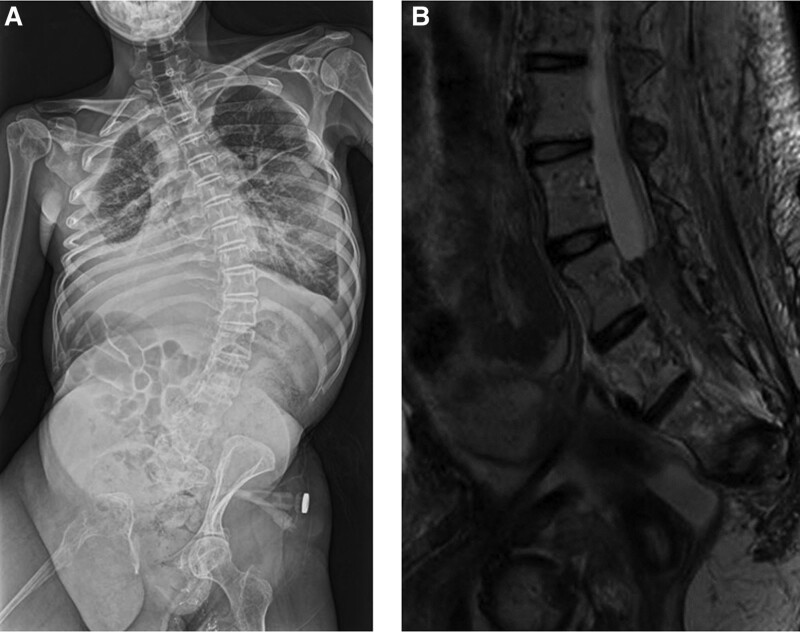
The whole-body radiograph shows massive osteolysis in the right femur, pelvic bone, sacrum, rib, and vertebrae (A). T2-weighted magnetic resonance imaging showing a low-iso intensity area of suspected hematoma from L3 to the caudal region (B).

At 22 years of age, she complained of headaches and severe pain in the hip and lower limb and was admitted to the hospital. The pain continued to worsen, and the patient often lost consciousness. The pain was confined to the lumbosacral region and the left side of the lower limb and head, but no osteolytic lesions were observed in the left limb (Fig. 1A). The pain was moderate, dull, and persistent, but sometimes breakthrough pain was spontaneous and intolerable. Computed tomography and magnetic resonance images revealed no recurrence of the chronic subdural hematoma. Additionally, an epidural hematoma extending from L3 to the caudal region was observed on magnetic resonance images (Fig. 1B). Laboratory test results were consistent with anemia and coagulopathy; hemoglobin 7.9 g/dL (normal 11.3–15.2 g/dL), platelet counts 244 × 10^9^/L (normal 158–358 × 10^9^/L), fibrinogen 144 mg/dL (normal 150–400 mg/dL), d-dimer 71.9 µg/mL (normal < 1 µg/mL).

We presumed that the pain was caused by intracranial hypotension due to minor CSF leakage and nerve root compression due to a lumbar epidural hematoma. After pregabalin was prescribed, rapid improvement was observed in the lower limb pain and headache. The patient experienced tolerable mild dizziness and somnolence. The lower limb pain worsened 2 months after treatment. However, this time, morphine was prescribed, which relieved the pain without any severe adverse events. Finally, oral administration of 120 mg morphine and 300 mg pregabalin daily helped her perform daily activities. However, the coagulopathy persisted, and the anemia and thrombocytopenia gradually progressed. She sometimes required transfusions of red blood cell products.

**Figure 2.**
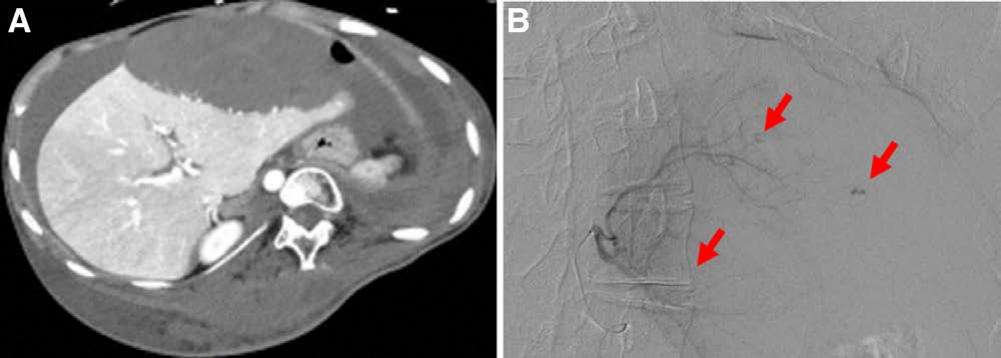
Computed tomography shows a massive hepatic subcapsular hemorrhage (A). Hepatic arteriography revealed a number of hemorrhage points (red arrow). However, no obvious hepatic arterial malformations were found (B).

Four months later, the patient suddenly presented with severe abdominal pain and went into shock with severe anemia. Computed tomography revealed a massive subcapsular liver hemorrhage (Fig. 2A), but no abnormal lymphangiomatous tissue was found in the liver. Emergency hepatic artery embolization was performed, and hemostasis was achieved (Fig. 2B).

## 3. Discussion

This report describes a young female patient with GSD who developed intractable headaches and pain in her hip and lower limb, probably caused by a lumbar epidural hematoma related to abnormal lymphangiomatous tissues. She also experienced sudden hepatic hemorrhage due to consumption coagulopathy caused by the progression of GSD.

The etiology and pathogenesis of GSD remain unknown. The main features of GSD are bone absorption and lymphangiogenesis. Osteoclasts play a crucial role in bone resorption. Humoral factors, such as interleukin-6, macrophage colony-stimulating factor, and receptor activator of NF-kappaB ligand, seem to promote osteoclastogenesis and the survival of osteoclasts.^[6,7,11]^ Furthermore, the augmented number of macrophage-like cells in GSD lesions produce VEGF-A, VEGF-C, and VEGF-D that can stimulate differentiation into osteoclasts and lymphangiogenesis.^[12]^ Conversely, some studies have suggested that bone resorption may be secondary to lymphangiogenesis. Growth factors secreted by the lymphatic endothelial cells may affect osteoclasts and osteoblasts. In GSD patients, it has been shown that lymphatic vascular endothelial hyaluronan markers are present on endothelial cells,^[13]^ and the levels of circulating platelet-derived growth factor-BB, which promotes lymphangiogenesis, are elevated.^[14]^ The endothelial cells express VEGF receptors, especially VEGF receptors-3, which is activated by VEGF-C, VEGF-A, and VEGF-D promoting angiogenesis and lymphangiogenesis in the bone marrow of GSD patients.^[15,16]^ As a result, bone resorption might be caused by mechanical compression of abnormal lymphangiomatous tissue.

Complaints in patients with GSDs are associated with osteolysis. The most common clinical presentation in a case series comprising 64 Chinese and 3 English patients included localized pain (n = 40; 59.7%).^[9]^ The pain can arise spontaneously or be caused by pathological fractures. Although all bones may be affected, the ribs, spine, and skull are frequently affected by GSD. The type of bone affected by the disease can negatively affect various daily activities. However, the present report showed that the painful lesion was not an osteolytic lesion; thus, the pain was probably not caused by osteolysis.

In patients with GSD, both lymphatic and vascular proliferations are observed in the osteolytic lesions. The abnormal lymphatic and blood vessel tissues easily collapse. Intrathoracic osteolytic lesions, such as those in the clavicle, rib, or thoracic vertebrae, can result in pleural effusions, including chylothoraces.^[3]^ Abnormal lymphangiomatous tissues proliferate and invade the surrounding tissues in the affected petrous bones, temporal bones, and vertebrae. Consequently, CSF leakage is caused by fistulae formation in the dura mater.^[17]^ CSF leakage can induce intracranial hypotension and lead to chronic subdural hematomas,^[18]^ as observed in this report. Furthermore, bleeding and hematoma have been reported in the affected regions following the injury of fragile proliferated lymphangiomatous tissues.^[19]^

Furthermore, consumption coagulopathy may occur in patients with GSD. It has been commonly reported that patients with lymphatic and venous malformations, such as complex lymphatic anomalies, have localized intravascular coagulopathy (LIC), which manifests as anemia, thrombocytopenia, the elevation of the D-dimer level, and coagulation dysfunction.^[20]^ Regarding GSD, 2 cases with serous effusion presented with LIC in a previous report.^[8]^ LIC might cause refractory serous effusions such as pleural effusion and bleeding. The presented report showed the features of LIC, an epidural hematoma, and subcapsular liver bleeding. From the above, the bleeding events in this report might have been caused by the combined factors of the vulnerability of abnormal lymphangiomatous tissues and LIC.

As shown in the current case, GSD often causes events related to bone vulnerability, including pain. Although infrequent in patients with GSD, bleeding associated with abnormal vascular disruption and coagulopathy can be life-threatening. Thus, we focused on symptoms other than those related to the affected bones in GSD.

## Acknowledgments

We would like to thank Editage (www.editage.com) for English language editing.

## Author contributions

**Conceptualization:** Yasutomo Kumakura, Norio Hasuda, Kazuki Akita.

**Data curation:** Yasutomo Kumakura.

**Supervision:** Tetsuya Iijima, Takashi Matsukawa.

**Writing – original draft:** Yasutomo Kumakura.

**Writing – review & editing:** Yasutomo Kumakura, Norio Hasuda, Kazuki Akita, Tetsuya Iijima, Takashi Matsukawa.
